# Predictors of Immediate Deterioration of the Child‐Pugh Classification From A to B After Transcatheter Arterial Chemo‐Embolization for Treatment‐Naive Hepatocellular Carcinoma

**DOI:** 10.1002/cam4.70367

**Published:** 2024-11-02

**Authors:** Kazuo Asano, Ken Kageyama, Akira Yamamoto, Atsushi Jogo, Mariko Nakano, Kazuki Murai, Yoshimi Yukawa‐Muto, Naoshi Odagiri, Kohei Kotani, Ritsuzo Kozuka, Etsushi Kawamura, Hideki Fujii, Sawako Uchida‐Kobayashi, Masaru Enomoto, Norifumi Kawada, Yukio Miki

**Affiliations:** ^1^ Department of Diagnostic and Interventional Radiology, Graduate School of Medicine Osaka City University Osaka Japan; ^2^ Department of Diagnostic and Interventional Radiology, Graduate School of Medicine Osaka Metropolitan University Osaka Japan; ^3^ Department of Hepatology, Graduate School of Medicine Osaka Metropolitan University Osaka Japan; ^4^ Department of Premier Preventive Medicine, Graduate School of Medicine Osaka Metropolitan University Osaka Japan

**Keywords:** Child‐Pugh classification, hepatocellular carcinoma, preserved liver function, transcatheter arterial chemoembolization

## Abstract

**Aim:**

The purpose of this study was to evaluate the predictors of deterioration of the Child‐Pugh classification 1 month after transcatheter arterial chemo‐embolization (TACE) in patients with treatment‐naive hepatocellular carcinoma (HCC).

**Methods:**

Between 2010 and 2020, consecutive patients who underwent conventional TACE using epirubicin as the initial treatment were enrolled. Patients with Barcelona Clinic Liver Cancer stage‐0, A or B and Child‐Pugh class A were included. The Child‐Pugh score was evaluated before treatment and 1 month after TACE. The following variables were analyzed by univariate and multivariate analyses as predictors of deterioration of the Child‐Pugh class from A to B: age, sex, etiology, serum albumin, bilirubin, prothrombin time (PT), encephalopathy, ascites, largest tumor diameter, tumor number, tumor location, α‐fetoprotein, protein induced by vitamin K absence or antagonist‐II, epirubicin dosage, ethiodized oil dosage, and number of treated liver segments.

**Results:**

A total of 152 patients were retrospectively enrolled. The deterioration rate of the Child‐Pugh class from A to B was 8.6%. Multivariable analysis showed that serum albumin ≤ 3.8 g/dL, PT ≤ 80%, and largest tumor diameter ≥ 3.8 cm were predictors of deterioration of the Child‐Pugh class. The deterioration rate to Child‐Pugh class B was 0% in patients with up to one of these factors, 14.3% in those with two factors, and 70% in those with three factors.

**Conclusions:**

A combination of serum albumin ≤ 3.8 g/dL, PT ≤ 80%, and largest tumor diameter ≥ 3.8 cm can predict the immediate deterioration of the Child‐Pugh classification from A to B following TACE.

## 1 Introduction

Treatment options for hepatocellular carcinoma (HCC) include ablation, resection, transplantation, transcatheter arterial chemoembolization (TACE), and systemic therapies [1, 2, 3, 4]. The treatment is primarily determined by the stage based on tumor burden, liver function, and physical status [4]. Recently, various molecular targeted agents (MTAs) and immune checkpoint inhibitors (ICIs) have shown promising results [5, 6, 7, 8]. Furthermore, there is increasing evidence of good outcomes with combination therapy using TACE and systemic therapy for patients with intermediate‐stage HCC [9, 10, 11]. The indications for systemic therapy are expanding to patients who were previously eligible for TACE treatment.

It is crucial for the patient to maintain Child‐Pugh class A for long‐term survival. Recent studies have suggested a reasonable correlation between Child‐Pugh class A and the safety and efficacy of systemic therapies [4, 12, 13]. Some studies showed that the combination therapy of TACE and MTAs significantly prolonged progression‐free survival and time to untreatable progression [10, 11]. These studies included patients with Child‐Pugh class A or Child‐Pugh score ≤ 7. When considering the combination of TACE and MTAs, it is critical that there is no deterioration of the Child‐Pugh classification in the short term. In the post‐TACE study, the combination of sorafenib and TACE did not significantly prolong time to progression [14]. In this previous study, the median time from last TACE to study randomization was 9.3 weeks (range: 5.6–13.3 weeks), concluding that the delay in starting sorafenib after TACE may have contributed to this poor prognosis. Later, in the TACTICS study, TACE plus sorafenib significantly prolonged progression‐free survival compared to TACE alone [10]. In this study, sorafenib was resumed 3 days after TACE, which may have contributed to the good results. Although the timing of MTA initiation after TACE remains controversial, it should be at least shorter than 9.3 weeks, the median in the post‐TACE study. In short, to maximize the effectiveness of the combination of TACE and systemic therapy, it is essential to preserve liver function, especially to maintain Child‐Pugh class A, immediately after TACE so that systemic therapy can begin immediately after TACE.

Several studies examined liver function changes after TACE in the last decade. However, to the best of our knowledge, no study has examined the risk factors for deterioration to Child‐Pugh class B within 9.3 weeks after TACE. Since deterioration to Child‐Pugh class B immediately after TACE delays the start of systemic therapy, it is essential to identify predictors of immediate deterioration of the Child‐Pugh classification after TACE in order to begin combination therapy. The purpose of this study was to evaluate the predictors of deterioration of the Child‐Pugh classification 1 month after TACE in patients with treatment‐naive HCCs.

## 2 Materials and Methods

### 2.1 Patients

Between January 2010 and December 2020, patients meeting the following criteria were included in the study: (1) new diagnosis of HCC according to the criteria of the Japan Society of Hepatology Guidelines [3]; (2) initial treatment with conventional TACE; (3) no subsequent conversion or combination therapy, such as ablation, resection, radiation, or systemic therapy within 3 months following the initial TACE; (4) laboratory tests performed 1 month post‐initial TACE; (5) classified as Barcelona Clinic Liver Cancer (BCLC) stage‐0, A or B; and (6) Child‐Pugh class A before the initial TACE. Patients who were still in Child‐Pugh class A 1 month after treatment were defined as the “Class A group,” and those who deteriorated to Child‐Pugh class B were defined as the “Class B group.” Our institutional review board approved this retrospective study (Approval number, 2021–265) and waived the requirement for patient consent for this retrospective review.

### 2.2 TACE Procedure

An interventional radiology computed tomography (CT) system (Nexaris Angio‐CT; Siemens Healthcare GmbH, Forchheim, Germany) or a C‐Arm dual‐phase cone‐beam CT system (Artis zee BA Twin; Siemens Healthcare GmbH) was used. For imaging, a nonionic iodine contrast agent (iohexol, Omnipaque 300 iodine, 300 mg I/mL; GE Healthcare, Tokyo, Japan) was used. The procedure was performed under local anesthesia using a 3‐Fr or 4‐Fr catheter and a microcatheter with a 1.7–2.1‐Fr tip. Digital subtraction angiography (DSA) of the celiac and common hepatic arteries, CT during hepatic arteriography (CTHA), and CT during arterial portography were performed to identify the tumor and its feeding arteries. Superselective or selective TACE was performed by advancing the catheter tip as close as possible to the tumor in the subsegmental or segmental arteries of identified feeders. An emulsion was prepared using a three‐way stopcock with a solution of epirubicin (Epirubicin; Nippon Kayaku Co. Ltd., Tokyo, Japan) and ethiodized oil (Lipiodol; Guerbet Japan Co. Ltd., Tokyo, Japan). Doses of epirubicin and ethiodized oil were determined by the operator based on tumor size, number of tumors, and liver function; the maximum dose for one TACE was 50 mg of epirubicin and 10 mL of ethiodized oil. After the emulsion was injected, 1‐ to 2‐mm gelatin sponge particles (Gelpart; Nippon Kayaku Co. Ltd.) were administered. The treatment endpoint was defined as loss of tumor staining on DSA.

### 2.3 Follow‐Up and Evaluations

Age, sex, etiology, serum albumin, bilirubin, prothrombin time (PT), encephalopathy, ascites, largest tumor diameter, tumor number, BCLC stage, tumor location, α‐fetoprotein, protein induced by vitamin K absence or antagonist‐II (PIVKA‐II), epirubicin dosage, ethiodized oil dosage, number of treated liver segments, Child‐Pugh score, albumin‐bilirubin (ALBI) score, and up‐to‐7 score were collected. All of the above factors were included in univariate analyses. On multivariate analysis, BCLC stage, Child‐Pugh score, ALBI score, and up‐to‐7 score were excluded to avoid linear dependence. Thus, age, sex, etiology, serum albumin, bilirubin, PT, encephalopathy, ascites, largest tumor diameter, tumor number, tumor location, α‐fetoprotein, PIVKA‐II, epirubicin dosage, ethiodized oil dosage, and number of treated liver segments were included in the multivariate analysis. Tumor location, number, and size were evaluated using preoperative CT or CTHA during the procedure. Based on the tumors' locations, patients were classified as the central group or the peripheral group; patients in the central group had any portion of the tumors within 1 cm of the main trunk or first branch of the portal vein, and patients with tumors outside this zone were classified as being in the peripheral group [15]. Etiology was classified as viral or nonviral according to laboratory tests and previous antiviral therapy. Ascites were evaluated by CT. The epirubicin dosage, ethiodized oil dosage, and the number of treated liver segments were obtained from the operative records. The Child‐Pugh score was evaluated before treatment and 1 month after TACE.

### 2.4 Statistical Analysis

Fisher's exact test was used to compare categorical variables, and the Mann–Whitney *U* test was used to compare continuous variables. Predictors of deterioration of the Child‐Pugh classification were investigated using multivariable logistic regression analysis with forward‐backward stepwise entry using the Akaike information criterion. Significance was determined by two‐tailed tests, with significance at a *p* < 0.05. The above analyses were performed using GraphPad Prism for Windows version 9.3.0 (GraphPad Software Inc., San Diego, CA, USA).

## 3 Results

### 3.1 Patient Characteristics

**FIGURE 1:**
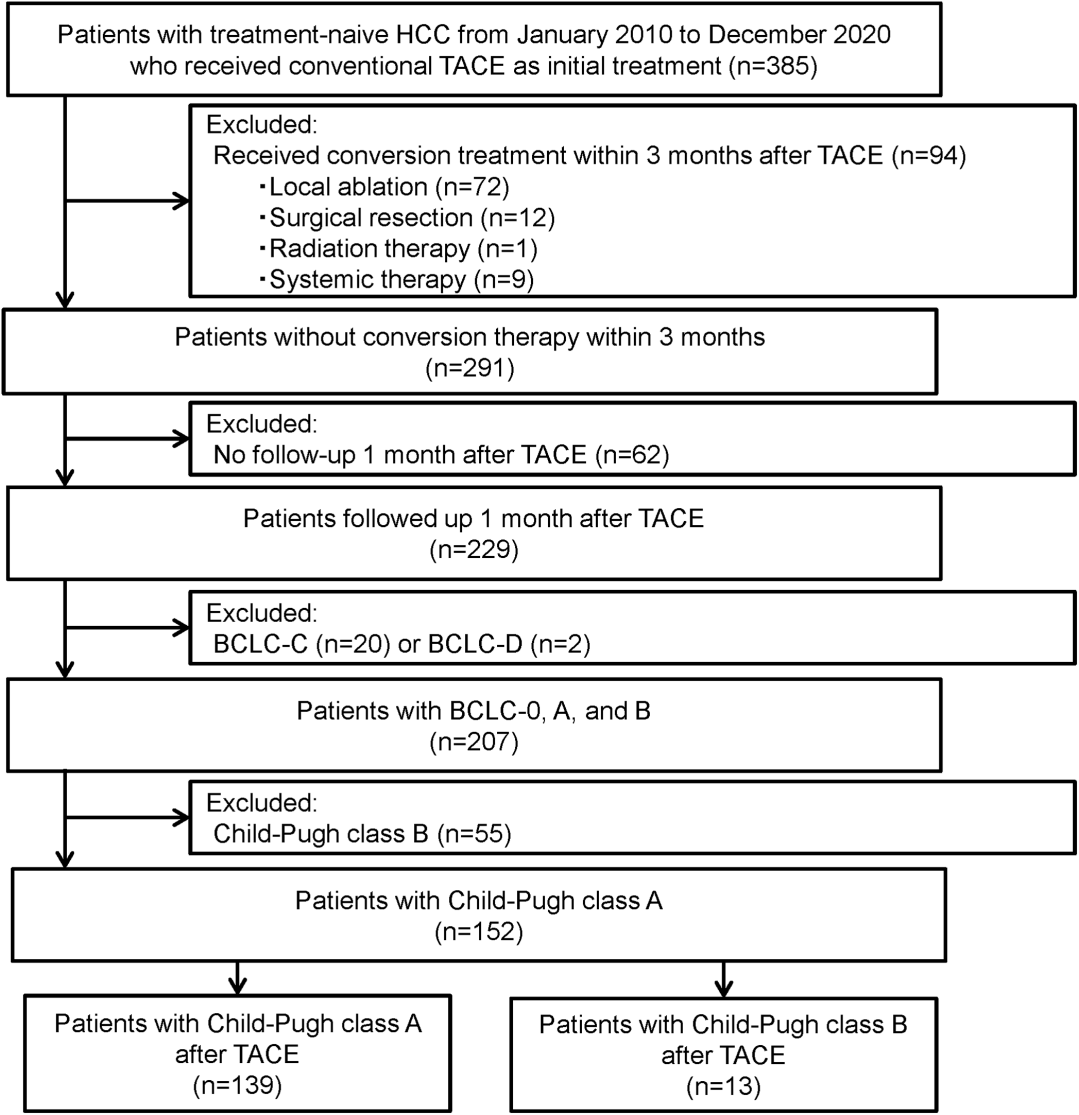
Flow diagram of patient selection. BCLC, Barcelona Clinic Liver Cancer; HCC, hepatocellular carcinoma; TACE, transcatheter arterial chemo‐embolization.

A total of 1225 treatment‐naive HCC patients who received treatment at our facility were retrospectively examined. Of them, 385 patients who received conventional TACE using epirubicin as the initial treatment were included. However, 230 patients were excluded for the following reasons: 85 patients received local conversion therapy; nine patients received systemic therapy within 3 months after TACE; 62 patients had no follow‐up 1 month after TACE; 20 patients were BCLC‐C; two patients were BCLC‐D; and 55 patients were Child‐Pugh class B. Consequently, 152 patients were enrolled in this study. Patients were classified by Child‐Pugh class after TACE as the Class A group (139/152, 91.4%) and the Class B group (13/152, 8.6%; Figure 1). The characteristics and univariate analyses of the patients are shown in Table 1. Age, sex, etiology, encephalopathy, and ascites were comparable. Serum albumin and PT% were higher in the Class A group than in the Class B group (3.9 ± 0.4 vs. 3.5 ± 0.3 g/dL, *p* < 0.001; 90.9 ± 13.9% vs. 72.7 ± 6.1%, *p* < 0.001, respectively). Serum bilirubin was lower in the Class A group (0.7 ± 0.3 vs. 1.1 ± 0.4 mg/dL, *p* < 0.001). Thus, the Child‐Pugh score and the ALBI score before TACE were significantly lower in the Class A group (5/6: 109/30 vs. 2/11, *p* < 0.001; −2.63 ± 0.37 vs. −2.11 ± 0.27, *p* < 0.001, respectively). Largest tumor diameter, tumor number, and up‐to‐7 score were lower in the Class A group (3.4 ± 1.9 vs. 4.0 ± 1.4 cm, *p* = 0.041; 4.7 ± 14.4 vs. 12.2 ± 26.7, *p* = 0.016; 8.1 ± 14.6 vs. 16.3 ± 26.7, *p* = 0.006, respectively). Thus, the Class B group had significantly more patients with BCLC‐B (0/A/B: 13/69/57 vs. 0/3/10, *p* = 0.039). PIVKA‐II was significantly higher in the Class A group (2792 ± 19,360 vs. 2161 ± 3294, *p* = 0.024), whereas the median was lower (120 vs. 942). There were no significant differences in α‐fetoprotein, tumor location, the doses of epirubicin and ethiodized oil, and the number of treated segments.

**TABLE 1:** Baseline characteristics and results of univariate analyses.

Characteristic	Overall (*n* = 152)	Child‐Pugh class A (*n* = 139)	Child‐Pugh class B (*n* = 13)	*p*
Age (year, mean ± SD)	72.4 ± 9.4	72.4 ± 9.5	72.6 ± 9.2	0.931
Sex (male/female)	111/41	100/39	11/2	0.516
Etiology (viral/non‐viral)	106/46	97/42	9/4	> 0.999
Albumin, g/dL (mean ± SD)	3.9 ± 0.4	3.9 ± 0.4	3.5 ± 0.3	< 0.001*
Bilirubin, mg/dL (mean ± SD)	0.8 ± 0.4	0.7 ± 0.3	1.1 ± 0.4	< 0.001*
PT, % (mean ± SD)	89.4 ± 14.3	90.9 ± 13.9	72.7 ± 6.1	< 0.001*
Encephalopathy (absent/present)	152/0	139/0	13/0	—
Ascites (absent/slight)	149/3	137/2	12/1	0.237
Child‐Pugh score (5/6)	111/41	109/30	2/11	< 0.001*
ALBI score (mean ± SD)	−2.58 ± 0.39	−2.63 ± 0.37	−2.11 ± 0.27	< 0.001*
Largest tumor diameter, cm (mean ± SD)	3.4 ± 1.9	3.4 ± 1.9	4.0 ± 1.4	0.041*
Tumor number (mean ± SD)	5.4 ± 15.8	4.7 ± 14.4	12.2 ± 26.7	0.016*
Up‐to‐7 score (mean ± SD)	8.8 ± 16.0	8.1 ± 14.6	16.3 ± 26.7	0.006*
BCLC stage (0/A/B)	13/72/67	13/69/57	0/3/10	0.039*
Tumor location (central/peripheral)	51/101	46/93	5/8	0.762
AFP, ng/mL (mean ± SD)	729 ± 3710	785 ± 3889	127 ± 213	0.117
PIVKA‐II, mAU/mL (mean ± SD)	2738 ± 18,471	2792 ± 19,360	2161 ± 3294	0.024*
Epirubicin, mg (mean ± SD)	32.5 ± 12.4	32.4 ± 12.6	33.4 ± 10.8	0.685
Ethiodized oil, ml (mean ± SD)	3.7 ± 1.7	3.6 ± 1.7	4.3 ± 1.7	0.106
Treated segment number (mean ± SD)	3.2 ± 2.0	3.1 ± 1.9	4.3 ± 2.6	0.081

Abbreviations: AFP, α‐fetoprotein; ALBI, albumin‐bilirubin; BCLC, Barcelona Clinic Liver Cancer; PIVKA‐II, protein induced by vitamin K absence or antagonist‐II; PT, prothrombin time; SD, standard deviation.

* *p* < 0.05.

### 3.2 Predictors of Deterioration of the Child‐Pugh Class

**FIGURE 2:**
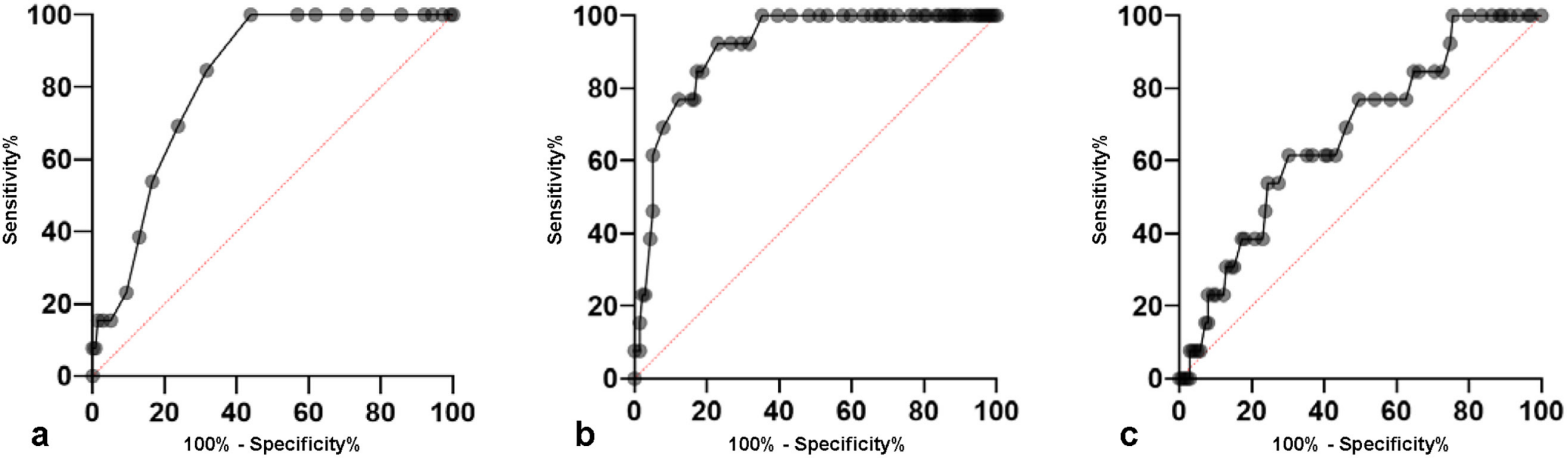
Receiver‐operating characteristic curve analyses. (a) Serum albumin: AUC 0.822, sensitivity 100%, and specificity 56.1% with a cutoff value of 3.8 g/dL, (b) Prothrombin time%: AUC 0.913, sensitivity 92.3%, and specificity 77% with a cutoff value of 80%. (c) Largest tumor diameter: AUC 0.671, sensitivity 61.5%, and specificity 69.8% with a cutoff value of 3.8 cm. AUC, area under the curve.

**FIGURE 3:**
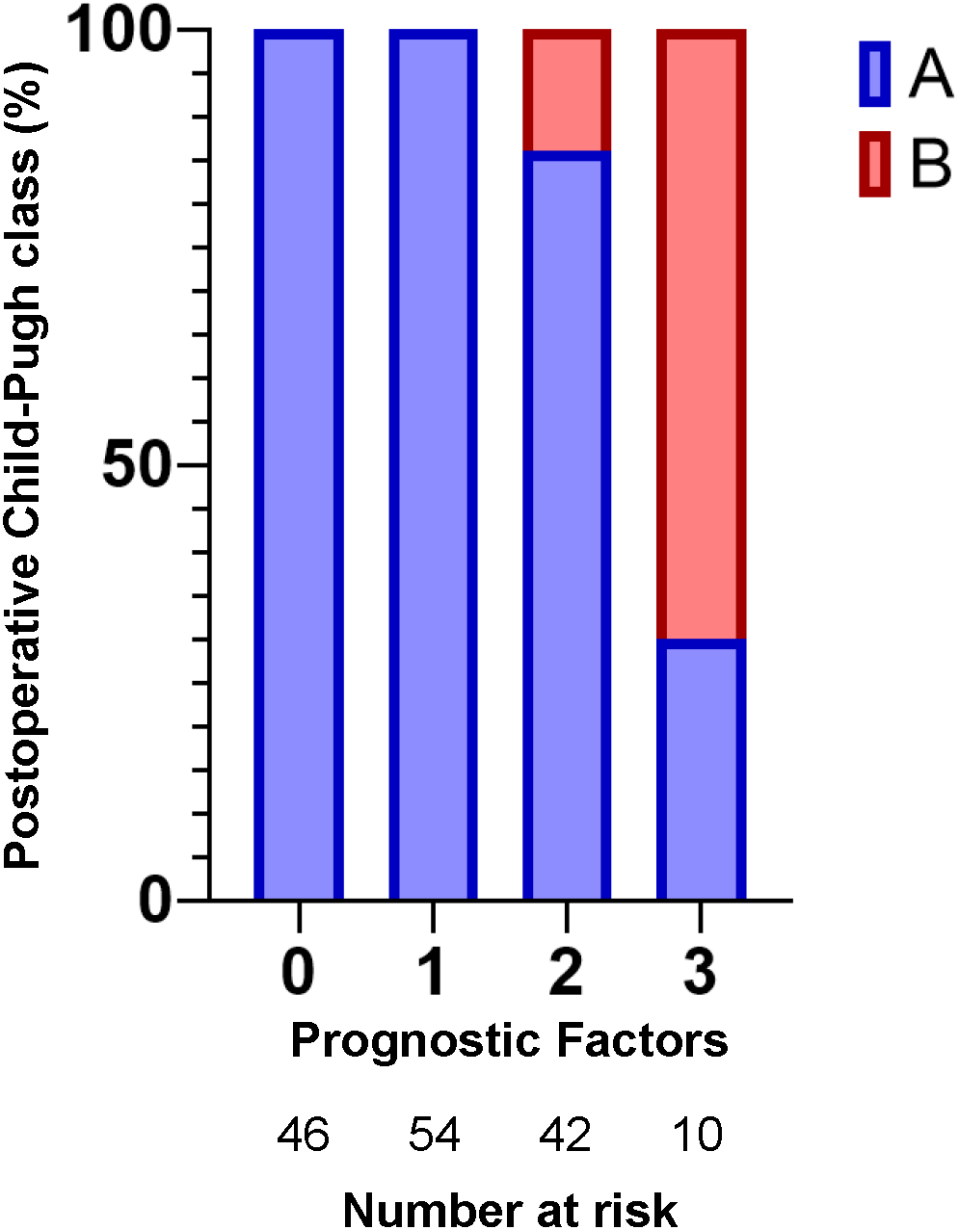
Child‐Pugh classification 1 month after TACE. The number of prognostic factors was calculated as the cumulative number of prognostic factors; that is, serum albumin ≤ 3.8 g/dL, prothrombin time ≤ 80%, and largest tumor diameter ≥ 3.8 cm. TACE, transcatheter arterial chemo‐embolization.

Serum albumin, bilirubin, PT%, ascites, and largest tumor diameter were selected for multivariable logistic regression analysis. Age, sex, etiology, encephalopathy, tumor number, tumor location, α‐fetoprotein, PIVKA‐II, epirubicin dosage, ethiodized oil dosage, and number of treated liver segments were not included in the multivariate analysis by stepwise entry. Serum albumin (odds ratio (OR) 0.005, *p* = 0.006), PT% (OR 0.80, *p* = 0.004), and largest tumor diameter (OR 1.99, *p* = 0.006) were significant predictors (Table 2). Receiver‐operating characteristic (ROC) curve analysis was performed for the three significant predictors. The analysis showed that the area under the curve (AUC) for serum albumin was 0.822, with a cutoff value of 3.8 g/dL, sensitivity of 100%, and specificity of 56.1% (Figure 2a). The AUC for PT% was 0.913, with a cutoff value of 80%, sensitivity of 92.3%, and specificity of 77% (Figure 2b). The AUC for the largest tumor diameter was 0.671, with a cutoff value of 3.8 cm, sensitivity of 61.5%, and specificity of 69.8% (Figure 2c). Thus, serum albumin ≤ 3.8 g/dL, PT ≤ 80%, and largest tumor diameter ≥ 3.8 cm appeared to be factors related to deterioration in the Child‐Pugh class. The deterioration rate to Child‐Pugh class B was 0% (0/100) in patients with up to 1 of these factors, 14.3% (6/42) in those with two factors, and 70% (7/10) in those with three factors (Figure 3).

**TABLE 2:** Multivariate logistic regression analysis.

Characteristic	Parameter estimate (95% CI)	Odds ratio (95% CI)	*p*
Albumin, g/dL (mean ± SD)	−5.38 (−10.1 to −2.17)	0.005 (0.00004–0.11)	0.006*
Bilirubin, mg/dL (mean ± SD)	2.66 (0.03 to 5.80)	14.36 (1.04–331.2)	0.060
PT, % (mean ± SD)	−0.23 (−0.41 to −0.10)	0.80 (0.66–0.91)	0.004*
Ascites (absent/slight)	3.89 (−0.004 to 8.39)	48.80 (0.99–4384)	0.053
Largest tumor diameter, cm (mean ± SD)	0.69 (0.23 to 1.27)	1.99 (1.26–3.56)	0.006*

Abbreviations: CI, confidence interval; PT, prothrombin time; SD, standard deviation.

* *p* < 0.05.

## 4 Discussion

This is the first study to identify the predictors of immediate deterioration of the Child‐Pugh class from A to B after TACE. This study evaluated liver function 1 month after TACE, which would be useful when considering the combination therapy of TACE and MTAs/ICIs. The rate of deterioration from Child‐Pugh class A to B after TACE for treatment‐naive HCC was 8.6%. Serum albumin ≤ 3.8 g/dL, PT ≤ 80%, and largest tumor diameter ≥ 3.8 cm were three independent predictors of Child‐Pugh class deterioration. In particular, the AUC for PT% was very high at 0.913, and it was a very valuable predictor. The AUC of serum albumin was second highest at 0.822, and it was also considered a sufficiently useful factor. Conversely, the AUC for maximum tumor diameter was not high at 0.671. However, tumor diameter was reported to be associated with patient prognosis and tumor hemorrhage in many previous studies, so it was very important to derive a threshold value for determining the treatment strategy [16, 17, 18, 19]. For patients with up to one of these factors, the rate of deterioration to Child‐Pugh class B was 0% in the present study. The deterioration rates in those with two and three factors were 14.3% and 70%, respectively. The combination of these factors had high predictive ability for deterioration from Child‐Pugh class A to B after TACE.

There is some international consensus that long‐term liver function worsens in patients who undergo TACE repeatedly despite TACE refractoriness [3, 20, 21]. It has been suggested that once TACE failure occurs, patients should switch to MTAs/ICIs immediately. It has also been proposed that patients should be considered for switching to MTA/ICI therapy earlier because it is sometimes too late to switch to MTA therapy after meeting the criteria of TACE refractoriness [3]. Lesions that are prone to cause deterioration of liver function in Child‐Pugh class B have been reported to be unsuitable for TACE [3]. Systemic therapies for TACE‐refractory HCC have been reported to be less likely to worsen liver function than TACE [21]. Taken together, these ideas support the earlier introduction of systemic therapy in patients whose liver function is likely to deteriorate to Child‐Pugh class B. Recently, with the demand for the combination therapy of TACE and MTAs/ICIs, it is important to maintain Child‐Pugh class A for the induction of MTAs/ICIs [3]. Therefore, it is critical to identify predictors that may worsen the Child‐Pugh class from A to B immediately after TACE.

Although many previous studies referred to the relationship between TACE and liver function, there have been few reports showing the predictors according to the deterioration of liver function after TACE in the short term (Table 3). The rate of deterioration from Child‐Pugh class A to B 1 month after TACE for treatment‐naive HCC was only 8.6% in the present study. The deterioration rate of the Child‐Pugh class from A to B 3 months after TACE was reported to be 9%–14% [22, 23]. Similar to the results at 3 months, the majority of cases maintained Child‐Pugh class A 1 month after TACE in the present study. Regarding short‐term liver enzyme changes and preserved function other than Child‐Pugh classification, large observational studies reported that the deterioration rate of laboratory values related to liver function within 90 days after TACE ranged from 11% to 52% [24, 25]. As for the ALBI score, we reported that the ALBI score decreased slightly 1 month after TACE, but it was not affected in the long‐term period of 3–6 months [15]. We also found no significant difference in the ALBI score before and 1–3 months after TACE following lenvatinib treatment in patients with previous TACE‐refractory HCC, even though TACE had been previously performed many times for the patients [26]. In terms of predictors, Takaya et al. showed that PT < 70% and ALBI score > −2.27 were significantly associated with deterioration of liver function from Child‐Pugh class A to B 3 months after TACE [23]. Yasui et al. reported that significant factors independently associated with deterioration to Child‐Pugh class B after TACE were exceeding the up‐to‐seven criteria, serum albumin ≤ 3.5 g/dL, serum bilirubin > 1.0 mg/ dL, and PT ≤ 80% at a mean observation period of 27.9 months [19]. Regarding the short‐term Child‐Pugh score, Kohla et al. reported that the percentage of patients with a worsening Child‐Pugh score 1 month after TACE was 52.9%, and that predictors for worsening included larger tumor size, higher serum α‐fetoprotein, and lower serum albumin [27]. However, this report had some limitations: it included patients with a history of previous TACE treatment, it did not consider the boundary value of the Child‐Pugh classification, and it did not show the cut‐off values of predictors. Thus, it is clinically impractical to use. The present study showed that serum albumin ≤ 3.8 g/dL and PT ≤ 80% were predictors of deterioration of the Child‐Pugh class from A to B at 1 month. These results were generally consistent with the results of previous reports. Whereas previous reports showed factors associated with deterioration of the Child‐Pugh class over a period longer than 3 months, the present study demonstrated predictors of deterioration of the Child‐Pugh class from A to B over a short‐term period of 1 month. This is shorter than the median time from TACE to sorafenib administration of 9.3 weeks in the post‐TACE study [14]. If TACE is initially performed in patients with low albumin and low PT%, subsequent scheduled systemic MTA/ICI therapies may be delayed or canceled, which may result in a worse prognosis. This result would be beneficial for planning the combination therapy of TACE and MTAs/ICIs.

**TABLE 3:** Summary of representative reported studies of liver function after TACE.

Study (year)	Research characteristics	Number of patients	Eligibility of patient	Time of evaluation after TACE	Evaluation of liver function	Deterioration of liver function	Factors for deterioration
Miksad et al. (2019) [24]	Multicenter retrospective study	572	HCC diagnosed within 1 year prior to the first TACE	Acute (0–29 days) Chronic (30–90 days)	Liver function laboratory values	Bilirubin: 30% and 23% Albumin: 52% and 31% AST: 44% and 25% ALT: 43% and 25% PT‐INR: 25% and 15%	
Peck‐Radosavljevic et al. (2018) [25]	International prospective study	1650	Unresectable HCCs; No prior systemic therapy	30–90 days	CTCAE grade	11%–29%	
Lao et al. (2011) [28]	Single‐center retrospective study	172	Child‐Pugh class A or B Hepatitis B surface antigen‐positive	30–90 days	3‐fold or greater increase in ALT or ALT > 100 IU/L Bilirubin > 41 μmol/L.	6.7%	
Takaya et al. (2021) [23]	Single‐center retrospective study	114.	Child‐Pugh class A Within up‐to‐seven criteria	3 months	Child‐Pugh class	12.7%	PT < 70% ALBI score > −2.27
Hiraoka et al. (2017) [22]	Single‐center retrospective study	212	BCLC stage B Child‐Pugh class A	3 months	Child‐Pugh class	9%–14%	
Park et al. (2020) [29]	Single‐center retrospective study	121	Newly diagnosed HCC Child‐Pugh class A	1 year	Child‐Pugh class	41.5%	Bilirubin > 0.82
Yasui et al. (2018) [19]	Single‐center retrospective study	224	Child‐Pugh class A	27.9 months (median)	Child‐Pugh class	1 year: 19.5% 3 years: 62.9% 5 years: 80.6%	Up‐to‐seven criteria Albumin ≤ 3.5 g/dL Bilirubin > 1.0 mg/dL PT ≤ 80%
Kohla et al. (2015) [27]	Single‐center prospective study	102	Child‐Pugh class A No prior locoregional therapy, systemic therapy, and/or surgical intervention	1 month	Child‐Pugh score	52.9%	Larger tumor size Higher serum AFP Lower serum albumin
Saito et al. (2012) [30]	Single‐center prospective study	109	Child‐Pugh class A or B	3 months	Child‐Pugh score	38.5%	Non‐protein respiratory quotient ratio Prealbumin ratio
Saito et al. (2012) [31]	Single‐center retrospective study	100	≥ 4 lesions or ≥ 3 cm HCCs Child‐Pugh class A or B	3 months	Child‐Pugh score (≥ 2 points)	11.0%	PIVKA‐II LDH
Sacco et al. (2009) [32]	Single‐center prospective study	71	≥ 4 lesions or ≥ 3 cm HCCs Child‐Pugh class A or B	5–7 days 4 months	Child‐Pugh score	Baseline: 5.62 ± 1.12 5–7 days: 6.11 ± 1.57 4 months: 5.81 ± 0.73	
Uchida‐Kobayashi et al. (2022) [26]	Single‐center retrospective study	68	TACE‐refractory HCC after lenvatinib treatment	1–3 months	ALBI score	Baseline: −2.37 1 month: −2.34 2 months: −2.24 3 months: −2.25	
Asano et al. (2023) [15]	Single‐center retrospective study	174	Treatment‐naive HCCs Child‐Pugh class A or B	1, 3, and 6 months	ALBI score	Baseline: −2.33 1 month: −2.22 3 months: −2.36 6 months: −2.46	

Abbreviations: ALBI, albumin‐bilirubin; ALT, alanine aminotransferase; AST, aspartate aminotransferase; BCLC, Barcelona Clinic Liver Cancer; CTCAE, common terminology criteria for adverse events; HCC, hepatocellular carcinoma; INR, international normalized ratio; LDH, lactate dehydrogenase; PT, prothrombin time; PIVKA‐II, protein induced by vitamin K absence or antagonist‐II; TACE, transcatheter arterial chemo‐embolization.

A greater largest tumor diameter was associated with the deterioration of the Child‐Pugh class from A to B in the present study, although the amount of epirubicin and ethiodized oil and the number of treated segments were not significant. This could mean that deterioration of the Child‐Pugh class was derived simply from tumor size rather than treatment intensity. Yasui et al. also reported that exceeding the up‐to‐7 criteria, which included the largest tumor diameter, was a significant factor in the deterioration of liver function [19]. Kim et al. reported that TACE for treatment‐naive HCC larger than 7 cm had a shorter prognosis and more frequent complications than for tumors smaller than 7 cm [16]. A high complication rate of TACE for large tumors contributed to the worsening nutritional status and liver damage, eventually leading to the deterioration of liver function. MTAs/ICIs for large HCCs also could cause major complications. We reported that lenvatinib therapy had a high risk of hemorrhage in large HCCs [18]. Combined therapy with atezolizumab and bevacizumab also induced bleeding in advanced HCC [17]. Thus, initial TACE for large HCCs would be beneficial for some patients to prevent tumor bleeding due to MTAs/ICIs. In the present study, patients with larger tumor diameters but high serum albumin and high PT% did not deteriorate to Child‐Pugh class B after TACE (Figure 3). For these patients, TACE might be performed prior to systemic MTA/ICI therapies to prevent tumor bleeding. However, patients with larger tumors, low serum albumin, and low PT% had high rates of Child‐Pugh class deterioration after TACE (Figure 3). In this situation, the patients cannot receive MTA/ICI therapies immediately after initial TACE due to Child‐Pugh class deterioration. Achieving a complete response for tumors with TACE alone might be difficult because of the large tumor size [16]. Overall, the combination with TACE and MTAs/ICIs is important to treat large‐sized HCCs. However, whether TACE or MTA/ICI therapies should be performed first remains debatable. Further studies are needed to determine which therapy should be given first in patients with large HCCs.

This study had limitations. This was a single‐center, retrospective study with a small number of cases. Notably, only 13 patients (8.6%) were included in the group that deteriorated to Child‐Pugh class B after TACE. Large prospective studies are needed to improve the validity of the results of this study. Second, since this study focused on treatment‐naive HCC patients, it is uncertain whether the same prognostic factors would apply to HCC patients with prior treatment histories. Third, this study focused on examining the association between identifiable factors before TACE and deterioration of the Child‐Pugh classification. Future studies should examine post‐TACE factors such as treatment efficacy and adverse events.

## 5 Conclusion

TACE for HCC in patients with serum albumin ≤ 3.8 g/dL, PT ≤ 80%, and largest tumor diameter ≥ 3.8 cm is likely to cause deterioration of the Child‐Pugh classification, leading to delayed introduction of systemic therapy. For such patients, alternative therapies might be considered to preserve their liver function and facilitate the introduction of subsequent treatment.

## Author Contributions

**Kazuo Asano:** conceptualization (supporting), data curation (lead), formal analysis (lead), investigation (lead), methodology (equal), visualization (equal), writing – original draft (lead), writing – review and editing (equal). **Ken Kageyama:** conceptualization (lead), data curation (equal), formal analysis (equal), investigation (lead), methodology (equal), resources (equal), supervision (equal), visualization (equal), writing – original draft (equal), writing – review and editing (equal). **Akira Yamamoto:** conceptualization (equal), methodology (equal), project administration (lead), resources (equal), supervision (equal), writing – original draft (equal), writing – review and editing (equal). **Atsushi Jogo:** investigation (equal), resources (equal), writing – review and editing (equal). **Mariko Nakano:** investigation (equal), resources (equal), writing – review and editing (equal). **Kazuki Murai:** investigation (equal), resources (equal), writing – review and editing (equal). **Yoshimi Yukawa‐Muto:** investigation (equal), resources (equal), writing – review and editing (equal). **Naoshi Odagiri:** investigation (equal), resources (equal), writing – review and editing (equal). **Kohei Kotani:** investigation (equal), resources (equal), writing – review and editing (equal). **Ritsuzo Kozuka:** investigation (equal), resources (equal), writing – review and editing (equal). **Etsushi Kawamura:** investigation (equal), resources (equal), writing – review and editing (equal). **Hideki Fujii:** investigation (equal), resources (equal), writing – review and editing (equal). **Sawako Uchida‐Kobayashi:** investigation (equal), resources (equal), writing – review and editing (equal). **Masaru Enomoto:** investigation (equal), resources (equal), writing – review and editing (equal). **Norifumi Kawada:** investigation (equal), resources (equal), writing – review and editing (equal). **Yukio Miki:** investigation (equal), supervision (equal), writing – review and editing (equal).

## Ethics Statement

The protocol for this research project was approved by a suitably constituted Ethics Committee of the institution (Committee of Osaka Metropolitan University, Approval No. 2021–265) that waived the requirement for patient consent for this retrospective review. It conforms to the provisions of the Declaration of Helsinki.

## Conflicts of Interest

The authors declare no conflicts of interest.

## Data Availability

The data that support the findings of this study are available on request from the corresponding author. The data are not publicly available due to privacy or ethical restrictions.
